# Supplementary material for: Insights into Halogen-Induced Changes in 4-Anilinoquinazoline EGFR Inhibitors: A Computational Spectroscopic Study
Source: Molecules. 2024 Jun 12;29(12):2800. doi: 10.3390/molecules29122800 (PMC11206398; doi:10.3390/molecules29122800)
Supplement: Supplementary file 1 [file molecules-29-02800-s001.zip › molecules-3016228-supplementary.pdf]

Insights into Halogen-Induced Changes in 4-Anilinoquinazoline EGFR Inhibitors: A Computational Spectroscopic Study

*Sallam Alagawani [1], Vladislav Vasilyev [2], Andrew H.A. Clayton [3,] * and Feng Wang [1,] **

[1]Department of Chemistry and Biotechnology, School of Science, Computing and Engineering Technologies, Swinburne University of Technology, Melbourne, Victoria 3122, Australia

[2]National Computational Infrastructure, Australian National University, Canberra, ACT 0200, Australia

[3]Optical Sciences Centre, Department of Physics and Astronomy, School of Science, Computing and Engineering Technologies, Swinburne University of Technology, Melbourne, Victoria 3122, Australia

*Correspondence: fwang@swin.edu.au (F.W); acalyton@swin.edu.au

Appendix

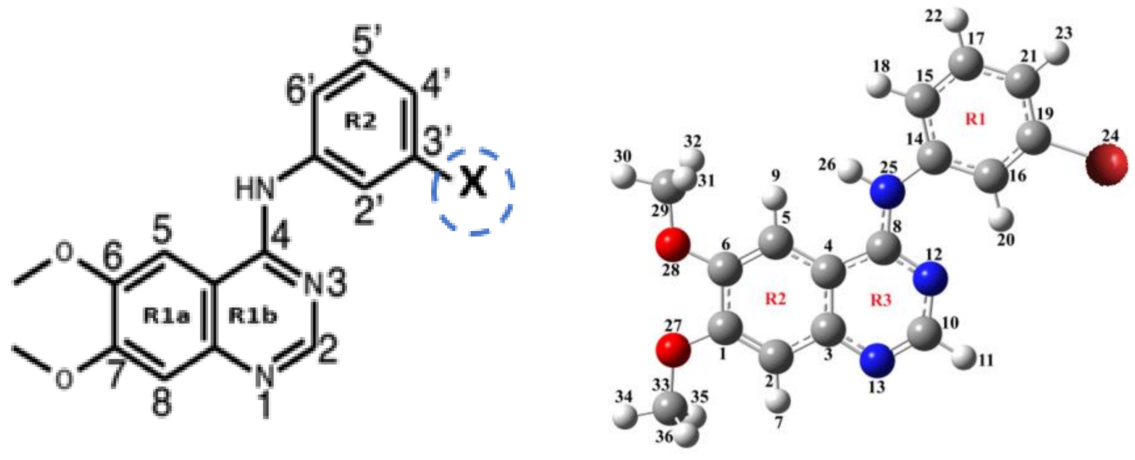

(a) (b)

Figure S1 The molecular composition of (a) TKIs-X, (b) PD153035 with (IUPAC name: N-(3-bromophenyl)-6,7-dimethoxyquinazolin-4-amine is provided. The numerical labels assigned to the atoms in the accompanying structural representation do not follow the IUPAC nomenclature but are utilized for ease of reference in Gaussian calculations, with the halogen atom (Br) being designated as atom 24.

Table S1.1 Molecular properties of the top five low energy TKIs-X conformers in DMSO solvent.

X	Property	1	2	3	4	5	EGFR-DB*
H	Δ*E* (Kcal mol^{-1})	0.000	2.720	3.474	3.478	3.710	1.346
	μ (Debye)	5.8286	4.9228	4.8242	4.8184	7.8611	5.8063
	%	89.665	0.840	0.230	0.228	0.153	8.880
	Structure	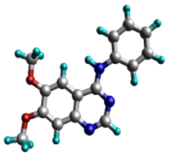	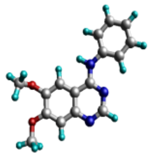	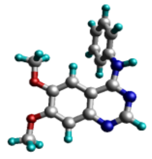	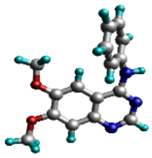	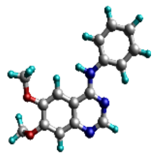	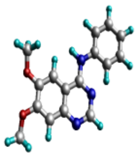
F	Δ*E* (Kcal mol^{-1})	0.000	0.540	2.776	3.340	3.753	0.876
	μ (Debye)	7.7915	4.5809	7.0239	4.6345	9.4848	5.9049
	%	61.211	24.209	0.521	0.198	0.097	13.603
	Structure	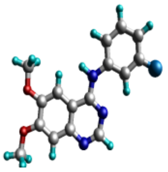	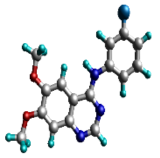	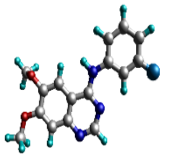	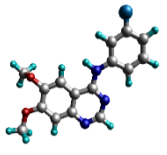	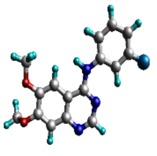	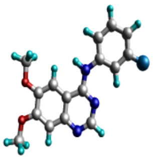
Cl	Δ*E* (Kcal mol^{-1})	0.000	0.341	2.784	3.145	3.765	8.193
	μ (Debye)	8.0755	4.4784	7.2823	4.6723	9.7130	0.7406
	%	63.493	35.373	0.532	0.286	0.099	0.001
	Structure	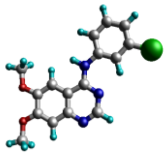	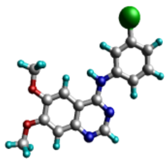	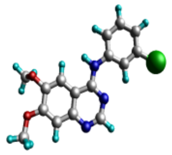	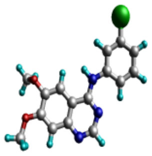	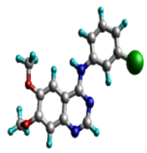	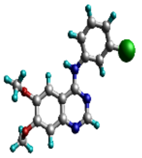
Br	Δ*E* (Kcal mol^{-1})	0.000	0.321	2.790	3.123	3.774	8.279
	μ (Debye)	8.2320	4.4362	7.4426	4.6323	9.8625	2.4071
	%	62.711	36.163	0.521	0.294	0.096	0.001
	Structure	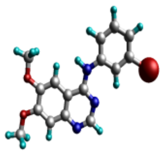	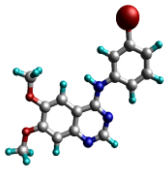	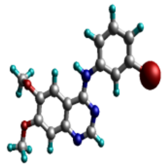	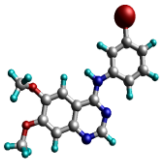	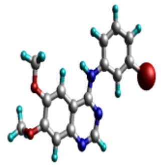	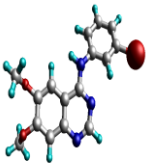
I	Δ*E* (Kcal mol^{-1})	0.000	0.256	2.768	3.030	3.773	1.040
	μ (Debye)	7.9890	4.5358	7.2109	4.6401	9.6185	5.8799
	%	54.619	35.160	0.470	0.300	0.084	9.156
	Structure	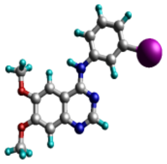	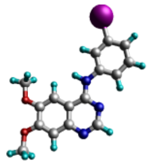	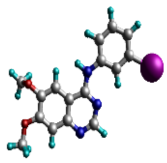	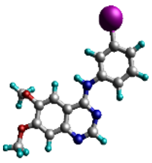	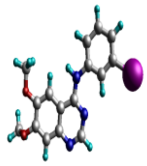	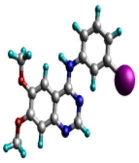

Table S1.2 Molecular properties of the five clustered lowest lying energy TKI-H conformers calculated using DFT B3LYP/def2TZVP method in DMSO solvent.

Conformer	Strain Energy (Kcal mol^{-1})	Dipole Moment (D)	Weight (%)
1	0.000	5.8286	89.665
2	2.720	4.9228	0.840
3	3.474	4.8242	0.230
4	3.478	4.8184	0.228
5	3.710	7.8611	0.153
EGFR-H	1.346	5.8063	8.880

Table S1.3 Molecular properties of the five clustered lowest lying energy TKI-F conformers calculated using DFT B3LYP/def2TZVP method in DMSO solvent.

Conformer	Strain Energy (Kcal mol^{-1})	Dipole Moment (D)	Weight (%)
1	0.000	7.7915	61.211
2	0.540	4.5809	24.209
3	2.776	7.0239	0.521
4	3.340	4.6345	0.198
5	3.753	9.4848	0.097
EGFR-F	0.876	5.9049	13.603

Table S1.4 Molecular properties of the five clustered lowest lying energy TKI-Cl conformers calculated using DFT B3LYP/def2TZVP method in DMSO solvent.

Conformer	Strain Energy (Kcal mol^{-1})	Dipole Moment (D)	Weight (%)
1	0.000	8.0755	63.493
2	0.341	4.4784	35.373
3	2.784	7.2823	0.532
4	3.145	4.6723	0.286
5	3.765	9.7130	0.099
EGFR-Cl	8.193	0.7406	0.001

Table S1.5 Molecular properties of the five clustered lowest lying energy TKI-Br calculated using DFT B3LYP/def2TZVP method in DMSO solvent.

Conformer	Strain Energy (Kcal mol^{-1})	Dipole Moment (D)	Weight (%)
1	0.000	8.2320	62.711
2	0.321	4.4362	36.163
3	2.790	7.4426	0.521
4	3.123	4.6323	0.294
5	3.774	9.8625	0.096
EGFR-Br	8.279	2.4071	0.001

Table S1.6 Molecular properties of the five clustered lowest lying energy TKI-I calculated using DFT B3LYP/def2TZVP method in DMSO solvent.

Conformer	Strain Energy (Kcal mol^{-1})	Dipole Moment (D)	Weight (%)
1	0.000	7.9890	54.619
2	0.256	4.5358	35.160
3	2.768	7.2109	0.470
4	3.030	4.6401	0.300
5	3.773	9.6185	0.084
EGFR-I	1.040	5.8799	9.156

Table S2 Total energy in Kcal mol^{-1} of the five clustered lowest lying energy TKI-X and EGFR crystal structures from database calculated using DFT B3LYP/def2TZVP in DMSO solvent.

Cluster (conformer)	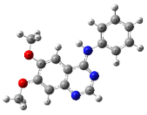 TKI-H	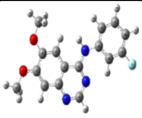 TKI-F	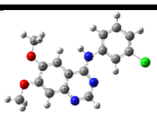 TKI-Cl	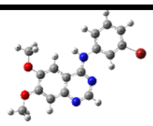 TKI-Br	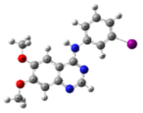 TKI-I
1	-933.798880	-1033.081728	-1393.425183	-3507.379134	-1230.996026
2	-933.794546	-1033.080867	-1393.424640	-3507.378623	-1230.995617
3	-933.793343	-1033.077304	-1393.420746	-3507.374688	-1230.991614
4	-933.793337	-1033.076406	-1393.420170	-3507.374157	-1230.991198
5	-933.792967	-1033.075747	-1393.419183	-3507.373119	-1230.990013
EGFR-DB*	-933.732360	-1033.007335	-1393.372796	-3507.325684	-1230.923945

*EGFR database (http://crdd.osdd.net/raghava/egfrindb/, H: EGIN0000732, F: EGIN0000733, Cl: EGIN0000281, Br: EGIN0000010, and I: EGIN0000736)

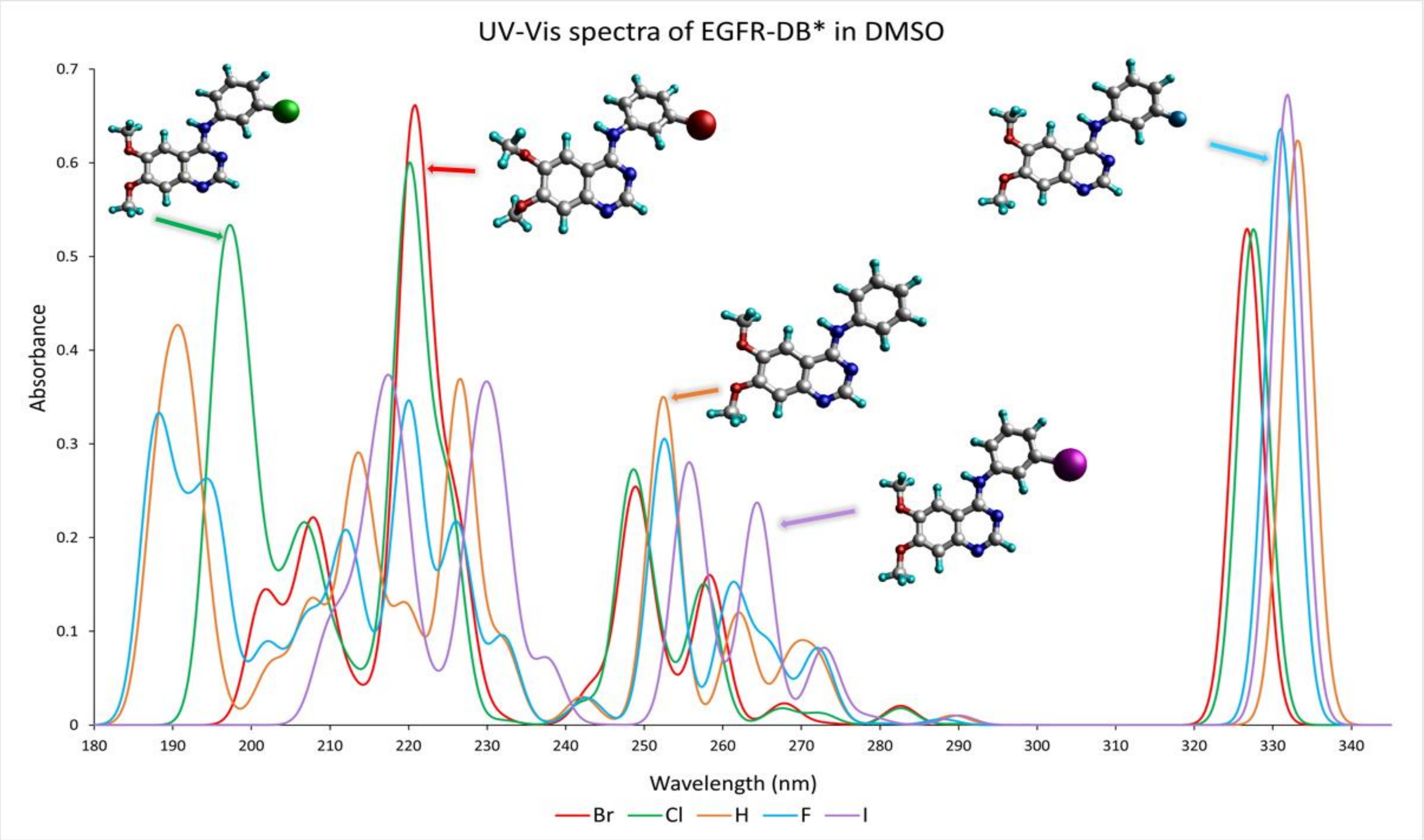

Figure S2.1 The UV-vis absorbance spectra (nm) of the EGFR* database crystal structures in DMSO solvent were calculated using the DFT B3LYP/def2TZVP method. EGIN0000732 is orange spectra, EGIN0000733 is blue spectra, EGIN0000281 is green spectra, EGIN0000010 is red spectra, and EGIN0000736 is purple spectra. *EGFR database (http://crdd.osdd.net/raghava/egfrindb/).

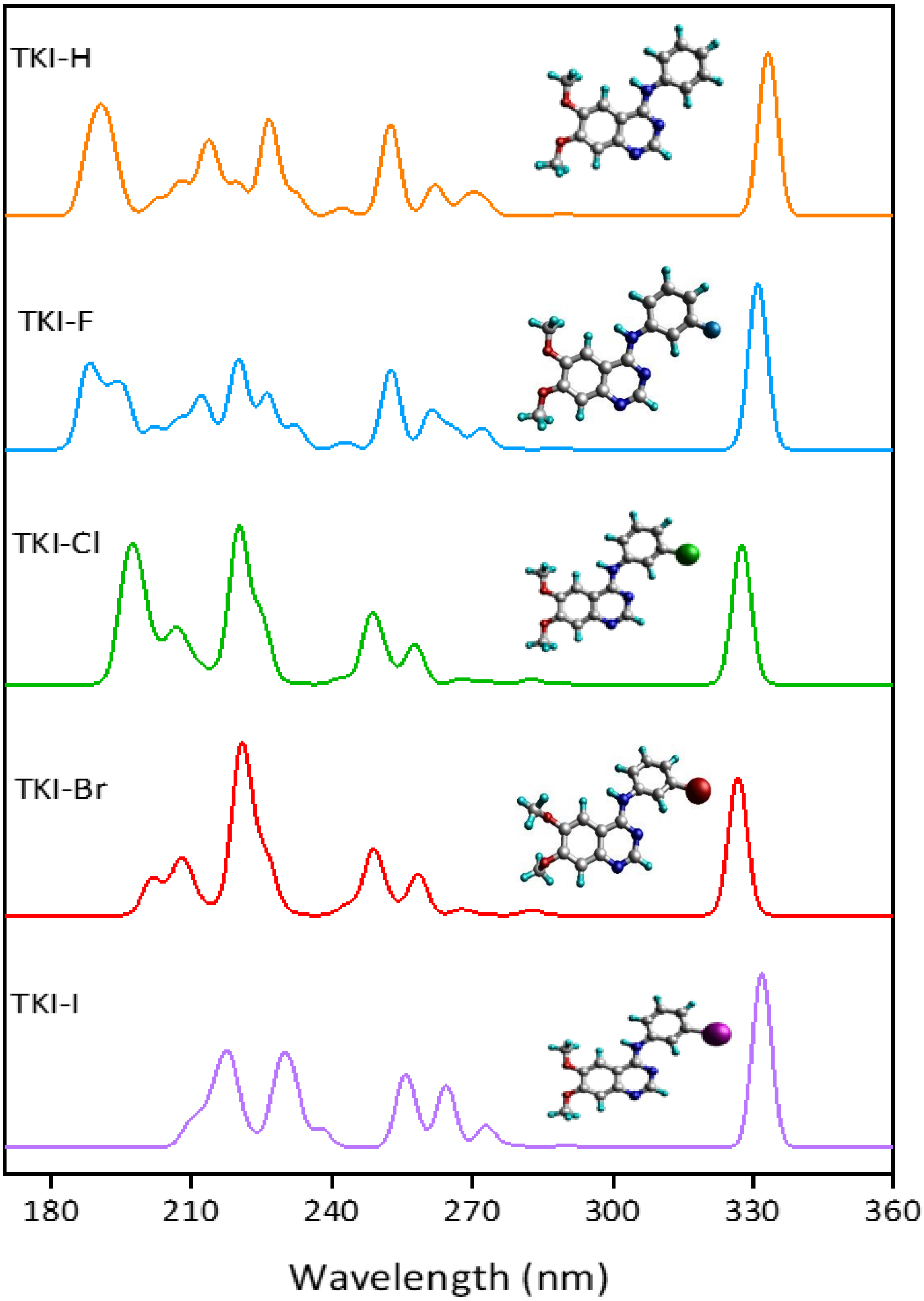

Figure S2.2 The UV-vis absorbance spectra (nm) of the halogen-substituted EGFR* inhibitors database crystal structures in DMSO solvent were calculated using the DFT B3LYP/def2TZVP method. EGIN0000732 is orange spectra, EGIN0000733 is blue spectra, EGIN0000281 is green spectra, EGIN0000010 is red spectra, and EGIN0000736 is purple spectra. *EGFR database (http://crdd.osdd.net/raghava/egfrindb/).

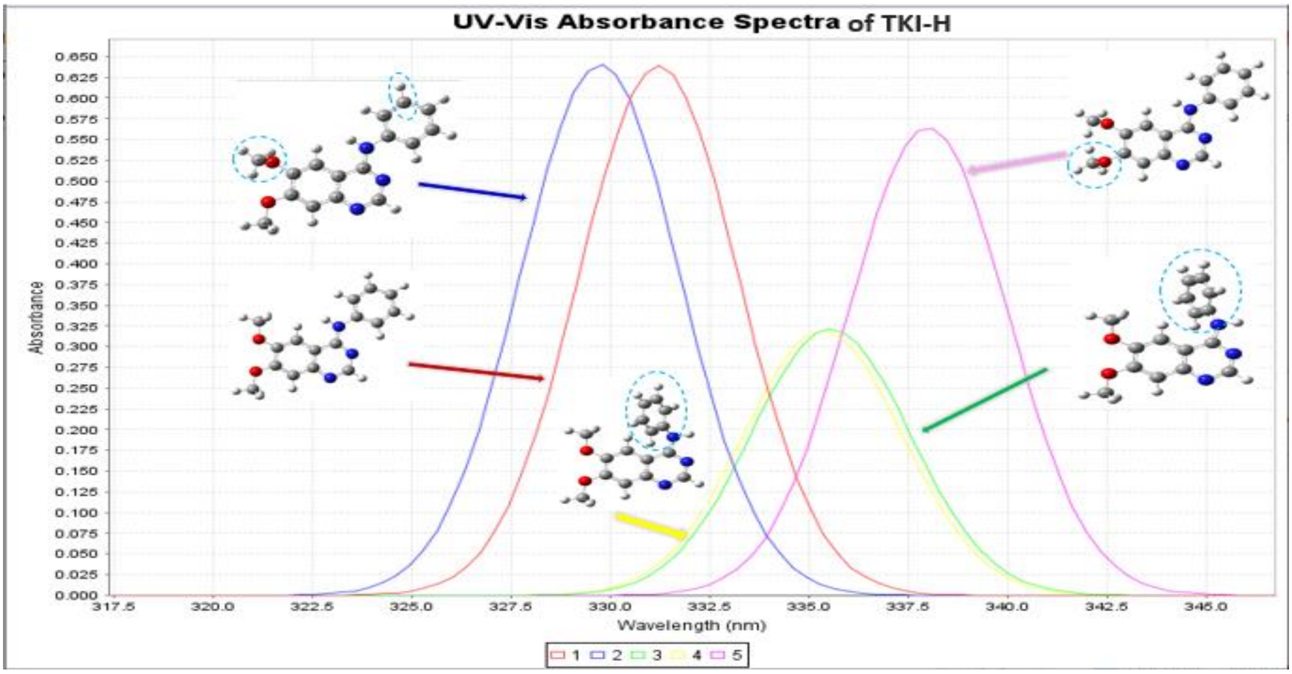

Figure S3.1 The UV-vis absorbance spectra (nm) of the first five low energy TKI-H conformers in DMSO were calculated using the DFT B3LYP/def2TZVP method. Conformers 1 and 2 contribute to 330 nm band, and conformers 3, 4, and 5 contribute to the band at 335.62 nm.

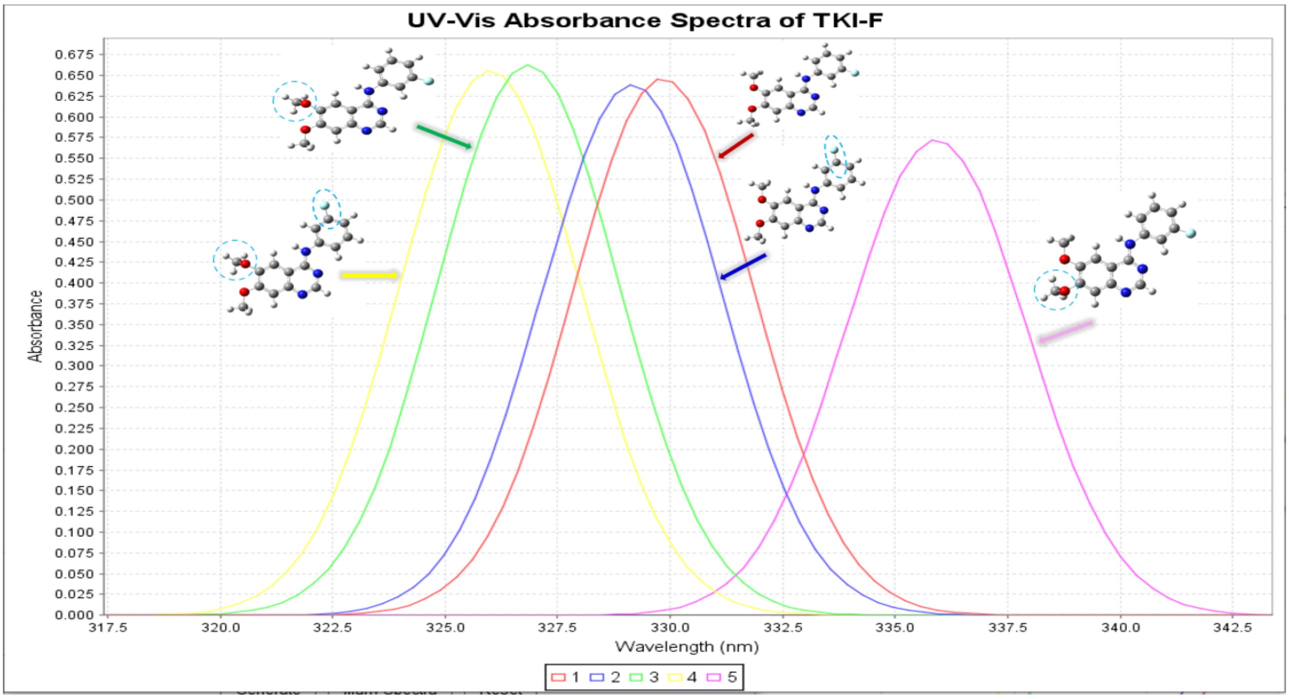

Figure S3.2 The UV-vis absorbance spectra (nm) of the first five low energy TKI-F conformers in DMSO were calculated using the DFT B3LYP/def2TZVP method. Conformers 1, 2, 3 and 4 contribute to 329.8 nm band, and conformer 5 contributes to the band at 335.91 nm.

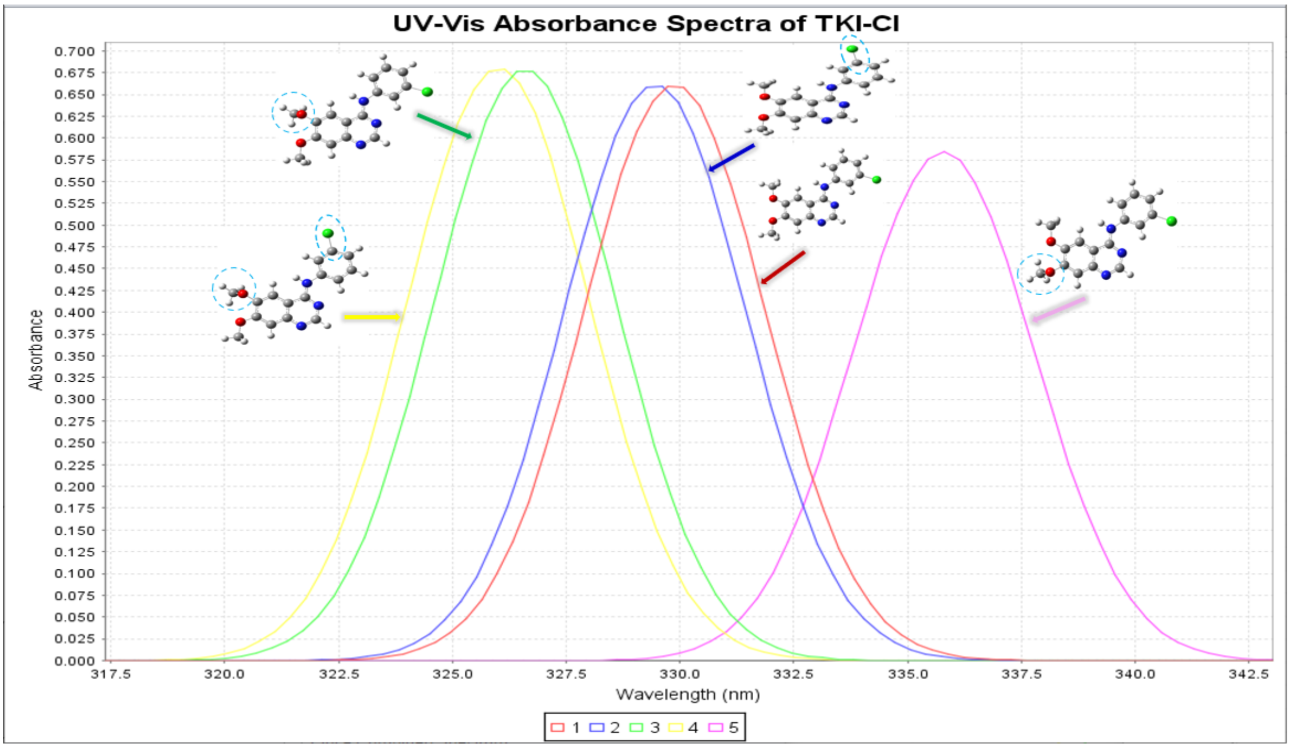

Figure S3.3 The UV-vis absorbance spectra (nm) of the first five low energy TKI-Cl (AG1478) conformers in DMSO were calculated using the DFT B3LYP/def2TZVP method. Conformers 1, 2, 3, and 4 contribute to 329.8 nm band, and conformer 5 contributes to the band at 335.79nm.

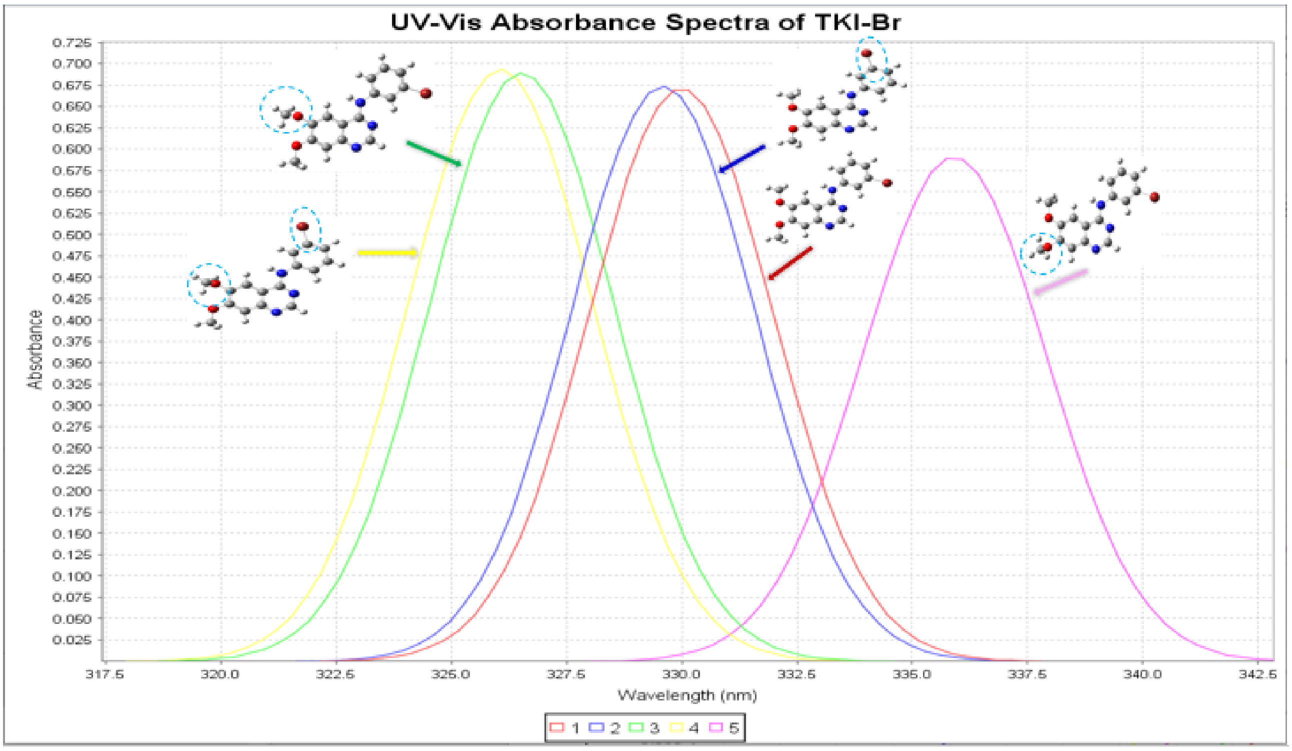

Figure S3.4 The UV-vis absorbance spectra (nm) of the first five low energy TKI-Br (PD153035) conformers in DMSO were calculated using the DFT B3LYP/def2TZVP method. Conformers 1 and 2 contribute to 328 nm band, and conformer 5 contributes to the band at 335.91 nm.

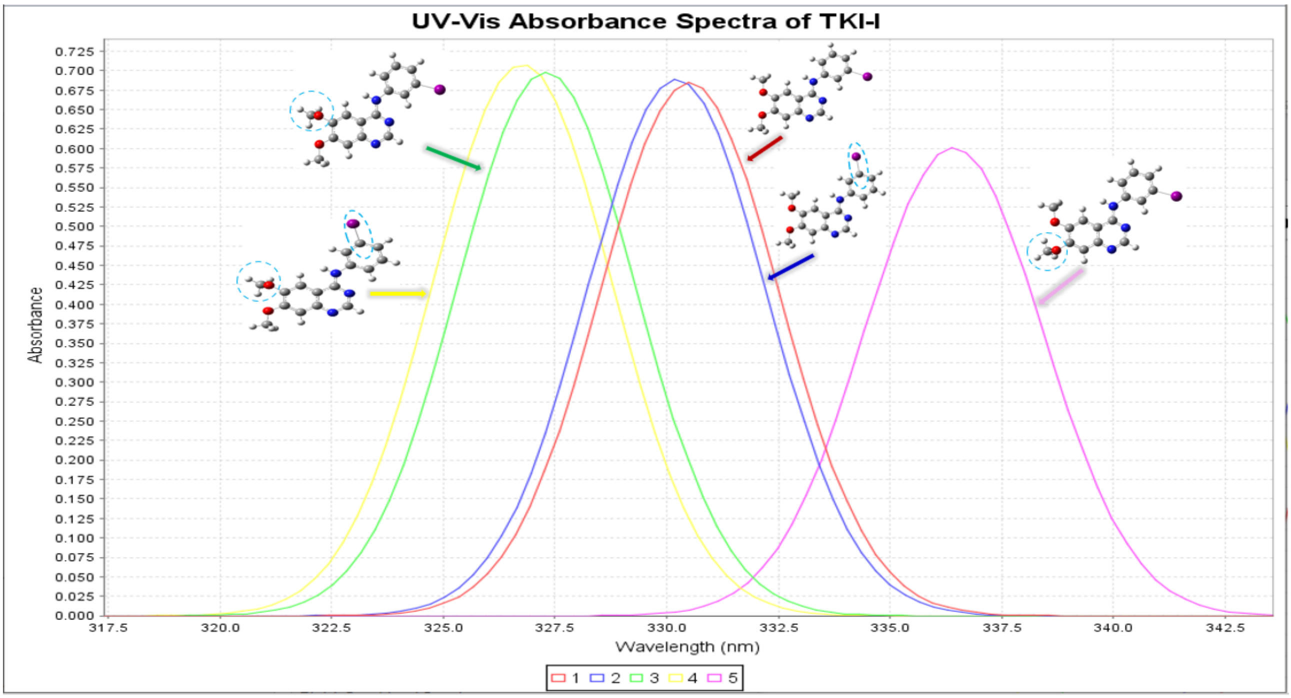

Figure S3.5 The UV-vis absorbance spectra (nm) of the first five low energy TKI-I conformers in DMSO were calculated using the DFT B3LYP/def2TZVP method. Conformers 1, 2, 3, and 4 contribute to 330 nm band, and conformer 5 contributes to the band at 336.42 nm.

Table S3 The calculated wavelength (nm) for the lowest energy absorption transition of TKI-X, employing the DFT B3LYP/def2TZVP model in DMSO solvent.

No.	H		F		Cl		Br		I	
	λ_{max}	f	λ_{max}	f	λ_{max}	f	λ_{max}	f	λ_{max}	f
1	331.21	0.64	329.85	0.65	329.88	0.66	329.99	0.67	330.51	0.69
2	329.74	0.64	329.15	0.64	329.45	0.66	329.59	0.67	330.22	0.69
3	337.99	0.56	326.85	0.66	326.6	0.68	326.53	0.69	327.31	0.7
4	335.54	0.32	325.99	0.66	326.01	0.68	326.07	0.69	326.77	0.71
5	335.62	0.58	335.91	0.57	335.79	0.58	335.91	0.59	336.42	0.6
**EGFR***	333.17	0.62	330.98	0.64	327.54	0.53	326.71	0.53	331.84	0.67

*EGFR database (http://crdd.osdd.net/raghava/egfrindb/, H: EGIN0000732, F: EGIN0000733, Cl: EGIN0000281, Br: EGIN0000010, and I: EGIN0000736). f is the oscillator strength value, λ_{max} is the absorption energy in (nm)

Table S4 Shifts in the maxima of absorption bands for transitions of EGFR-DB* structures of TKIs-X possessing oscillator strengths greater than 0.22 and electronic transition configurations exceeding 5% [a]

**H**			**F**			**Cl**			**Br**			**I**		
**λ (nm)**	***f***	**Major contributes**	**λ (nm)**	***f***	**Major contributes**	**λ (nm)**	***f***	**Major contributes**	**λ (nm)**	***f***	**Major contributes**	**λ (nm)**	***f***	**Major contributes**
333.17	0.6249	H→L (49%)	330.98	0.6369	H→L (49%)	327.54	0.5294	H→L (49%)	326.71	0.5295	H→L (49%)	331.84	0.6728	H→L (49%)
252.43	0.3507	H→L+3 (43%)	252.56	0.3057	H-4→L (5%)	220.04	0.5732	H-2→L+1 (5%)	220.43	0.5542	H-9→L (13%)	264.36	0.2372	H-3→L (14%)
226.53	0.3676	H-2→L+1 (29%)			H→L+2 (9%)			H-1→L+1 (7%)			H-1→L+2 (14%)			H-2→L (6%)
191.30	0.2861	H-4→L+2 (18%)			H→L+3 (27%)			H-1→L+2 (17%)						H-1→L (12%)
		H-4→L+3 (11%)	220.04	0.3387	H-2→L+1 (9%)	96.11	0.3351	H-7→L (6%)						H→L+2 (13%)
		H-1→L+4 (11%)			H-2→L+2 (10%)			H→L+4 (6%)				255.43	0.2459	H→L+4 (41%)
					H-1→L+2 (20%)			H→L+6 (9%)				218.50	0.2520	H-6→L (5%)
			187.97	0.2468	H-7→L+1 (11%)									H-2→L+3 (5%)
					H-2→L+5 (12%)									H-1→L+4 (27%)
														H→L+5 (5%)

[a]B3LYP/def2TZVP method in DMSO solvent. λ: denotes excitation energy, *f*: represents oscillator strength. *EGFR database (http://crdd.osdd.net/raghava/egfrindb/, H: EGIN0000732, Cl: EGIN0000733, F: EGIN0000281, Br: EGIN0000010, and I: EGIN0000736) [17].

Table S5 Shifts in the maxima of absorption bands for transitions of the global minimum structures of TKIs-X possessing oscillator strengths greater than 0.22 and electronic transition configurations exceeding 5% [a]

**H**			**F**			**Cl**			**Br**			**I**		
**λ(nm)**	***f***	**Major contributes**	**λ(nm)**	***f***	**Major contributes**	**λ(nm)**	***f***	**Major contributes**	**λ(nm)**	***f***	**Major contributes**	**λ(nm)**	***f***	**Major contributes**
331.21	0.6391	H→L (49%)	329.85	0.6462	H→L (49%)	329.88	0.6611	H→L (49%)	329.99	0.6710	H→L (49%)	330.51	0.6853	H→L (49%)
212.66	0.2902	H-2→L+2 (14%)	252.33	0.2985	H-4→L (5%)	254.54	0.2787	H→L+3 (42%)	254.94	0.2759	H→L+3 (42%)	263.62	0.2370	H-3→L (14%)
		H-1→L+3 (12%)			H→L+2 (7%)	197.55	0.2434	H-5→L (13%)	199.58	0.2778	H-6→L (15%)			H-2→L (6%)
		H→L+4 (13%)			H→L+3 (29%)			H-3→L+2 (5%)			H-3→L+3 (12%)			H-1→L (12%)
191.06	0.2696	H-4→L+2 (15%)	186.99	0.2744	H-7→L (5%)			H-3→L+3 (6%)			H-1→L+5 (7%)			H→L+2 (12%)
		H-4→L+3 (13%)			H-4→L+3 (6%)			H-1→L+4 (11%)			H→L+5 (5%)	255.25	0.2305	H→L+4 (41%)
		H-1→L+4(13%)			H-2→L+4 (17%)	216.50	0.3059	H-2→L+1 (5%)	216.71	0.3730	H-1→L+3 (28%)	218.03	0.2429	H-2→L+3 (6%)
252.15	0.3315	H→L+2 (43%)			H-1→L+4 (8%)			H-2→L+2 (8%)			H→L+5 (10%)			H-1→L+4 (23%)
226.14	0.3486	H-2→L+1 (28%)	219.93	0.3442	H-2→L+1 (9%)			H-1→L+3 (21%)						
					H-2→L+2 (8%)									
					H-1→L+2 (21%)									

[a]B3LYP/def2TZVP method in DMSO solvent. λ: denotes excitation energy, *f*: represents oscillator strength.
